# Metal Allergy Mediates the Development of Oral Lichen Planus via TSLP-TSLPR Signaling

**DOI:** 10.3390/jcm11030519

**Published:** 2022-01-20

**Authors:** Mohammad Fadyl Yunizar, Megumi Watanabe, Lipei Liu, Norikazu Minami, Tetsuo Ichikawa

**Affiliations:** Department of Prosthodontics & Oral Rehabilitation, Graduate School of Biomedical Sciences, Tokushima University, 3-18-15, Kuramoto, Tokushima 770-8504, Japan; c301851006@tokushima-u.ac.jp (M.F.Y.); c301751017@tokushima-u.ac.jp (L.L.); minamino@tokushima-u.ac.jp (N.M.); ichi@tokushima-u.ac.jp (T.I.)

**Keywords:** metal allergy, oral lichen planus, TSLP-TSLPR

## Abstract

Metal allergy is a T-cell-mediated delayed type of hypersensitive reaction. The pathogenetic mechanisms underlying the allergy are unclear, although the condition has been reported to be related to oral lichen planus (OLP), despite an absence of immunological studies to support this relationship. In this study, histopathological samples of OLP patients were examined to compare the metal allergy-positive and -negative groups, with a focus on the network of epidermal keratinocytes and T cells induced by thymic stromal lymphopoietin (TSLP) and its receptor, TSLPR. Infiltration of T cells into the epithelium was revealed to be higher in the OLP lesions of metal allergy-positive patients than in those of metal allergy-negative patients. Moreover, TSLP-TSLPR signaling and TNF-α production were higher in the epithelial tissue samples of the metal allergy-positive patients than in the metal allergy-negative patients. Metal allergy is associated with both increased expressions of TSLP in keratinocytes and increased TNF-α levels in the epithelium. We propose that this would promote the accumulation of T cells at the lesion site, contributing to the formation of the disease. These results suggest that metal allergy may be an aggravating factor in the pathogenesis of OLP.

## 1. Introduction

Metal allergy is a delayed type of hypersensitive reaction, the pathogenesis of which is mediated by a complex immune network of T-cell-mediated interactions [1,2,3]. However, the precise mechanisms by which metal allergy causes disease have not been fully elucidated. Although skin diseases such as palmoplantar pustulosis and psoriasis have been considered to be related to metal allergy [4,5,6], their causal roles in the development of metal allergy have not yet been established. Among similar skin diseases, oral lichen planus (OLP) has been posited to be related to dental metal allergy [7]. During dental treatment, symptoms of OLP can be observed on the oral mucosa in areas where it is in contact with metal. Furthermore, it is common for such patients to test positive for allergies to some metals upon analysis with a patch test. From epidemiological and clinical perspectives, dentists have suggested that there may therefore be a relationship between metal allergy and OLP, but to date, no immunological studies have been conducted to test this hypothesis. As both metal allergy and OLP are caused by the presence of T cells in the skin and mucosa, we consider it vital to focus on the network of interactions between T cells and keratinocytes to determine the relationship, if any, between these two diseases.

In this study, the histopathology details of patients diagnosed by biopsy as having OLP were examined by comparing differences between metal allergy-positive and -negative groups. The network of interactions between epidermal keratinocytes and T cells induced by TSLP and its receptor TSLPR were then analyzed for differences.

## 2. Materials and Methods

### 2.1. Patients

A total of 64 patients, 12 males and 52 females, who visited the Department of Oral Surgery and the Department of Oral Medicine at Tokushima University Hospital, were diagnosed with OLP by biopsy. Of these patients, 34 patients, 6 males and 28 females, were diagnosed as positive for metal allergy, and 30 patients, 6 males and 24 females, were diagnosed as negative for metal allergy by patch testing. All mucosal tissue samples were excised prior to the patch test, with metal still being present in the mouth at this point. Immunohistochemical staining was performed to screen for immune cell populations that had infiltrated the oral mucosa, and the tissues of patients diagnosed with OLP who were allergic to some metals (as shown by patch tests) were compared to tissues of those who were not allergic to metals.

### 2.2. Antibodies

The primary antibodies specific for human UCHL1, S-100 and cytokeratin were obtained from NICHIREI BIOSCIENCE INC. (Tokyo, Japan). Antibodies specific for human CD11b were obtained from BioLegend, Inc. (San Diego, CA, USA). The antibody specific for human TNF alpha was from eBioscience (San Diego, CA, USA). Antibodies specific for human TSLP and TSLPR were obtained from R&D SYSTEMS, Inc. (Minneapolis, MN, USA).

### 2.3. Histology and Immunohistochemistry

Paraffin-embedded blocks made for diagnosis by biopsy were used. The samples were cut into 8 μm slices using a microtome REM-710·SB (YAMATO KOUKI, Miyazaki, Japan) and placed on MAS-coated glass slides (Matsunami Glass IND LTD, Osaka, Japan). Sections were stored at room temperature until they were used. Dewaxing was performed in xylene (FUJIFILM Wako Pure Chemical Corporation, Osaka, Japan) for 10 min, followed by rinsing in 90%, 95%, and 100% ethanol (Wako) for 10 min each. The sections were treated with 100% methanol (Wako) containing 0.3% H_2_O_2_ (Wako) for 10 min to block endogenous peroxidase activity and thereby reduce non-specific background staining. The sections were incubated overnight at 4 °C with primary antibodies. After two 10-min washes, Histofine simple stain regent (NICHIREI) was applied as an HRP antibody for 30 min at room temperature, followed by two washing steps of 10 min. Sections were incubated with DAB (NICHIREI) for a time that achieved optimal coloration. Sections were then incubated for 30 s in hematoxylin (MUTO PURE CHEMICAL, Tokyo, Japan) for nuclear counter staining and placed in running tap water for 10 min. Sections were then dehydrated by being dipped in 80%, 90% and 100% ethanol and dipped in xylene twice. Finally, sections were covered with the mounting medium Entellan^®^ new (Merck, Darmstadt, Germany).

### 2.4. Analysis of Immunohistochemistry Staining

Immunohistochemistry images were taken by a Keyence All-in-One Fluorescence Microscope (BZ-X800, Osaka, Japan). The resultant 100× magnification image was analyzed by a Keyence Software, BZ-X800 Analyzer. The tissue area was separated into epithelial and subepithelial areas with a border line. The percentage of the cell number was calculated by the total area of cell spreading divided by the total area of the tissue (Supplementary Materials Figure S1).

### 2.5. Cell Culture

Human keratinocyte cell line HaCaT was kindly provided by Dr. Y. Kudo (Tokushima University, Tokushima, Japan). The cells were cultured in Dulbecco’s Modified Eagle Medium (DMEM) (Nacalai Tesque, Kyoto, Japan) supplemented with 10% fetal bovine serum (FBS) and 1% penicillin/streptomycin/amphotericin B in a humidified atmosphere of 5% CO_2_ at 37 °C. HaCaT cells were stimulated with 250 μM NiCl_2_ for 24, 48, and 72 h before analysis.

### 2.6. Quantitative Real-Time PCR Reactions and Primers

Real-time polymerase chain reaction (PCR) was performed to screen for pro-inflammatory cytokines produced by human epithelial cells in response to metal stimulation. The expression of inflammatory cytokines in HaCaT upon stimulation with the metal antigen NiCl_2_ was then analyzed. Total RNA was extracted using TRIzol (Thermo Fisher Scientific, Waltham, MA, USA) according to the manufacturer’s protocol. First-strand cDNA was synthesized with 500 ng RNA by PrimeScript (Takara bio Inc., Kusatsu, Japan). The real-time PCR was performed with TB-Green (Takara bio Inc.) on the ABI7300 Real Real-time PCR System (Applied Biosystems, Waltham, MA, USA) according to manufacturer instructions. The primer sequences are as follows.

TNF-α sense: 5′-AGCCTGTAGCCCATGTTGTAGC-3′, antisense: 5′-ATCTCTCAGCTCCACGCCATTG-3′, IL-1β sense: 5′-GCTGAGGAAGATGCTGGTTC-3′, antisense: 5′-GTGATCGTACAGGTCGCATCG-3′, TSLP sense: 5′-GCCATGAAAACTAAGGCTGC-3′, antisense: 5′-CGCCACAATCCTTGTAATTG-3′, TSLPR sense: 5′-TGACGTGTTCTGACCTGTCC-3′, antisense s: 5′-TCCATAGCCTTCACCCTGAC-3′, CCR2 sense: 5′-GGCCTGATAACTGTGAAAGCA-3′, antisense: 5′-CAGCAGTGAGTCATCCCAAGAG-3′, CCL2 sense: 5′-CAAGTGTCCCAAAGAAGCTG-3′, antisense: 5′-GAGTTTGGGTTTGCTTGTCC-3′, IL-6 sense: 5′-TGAGGAGACTTGCCTGGTGA-3′, antisense: 5′-GTTGGGTCAGGGGTGGTTAT-3′, IL-12 sense: 5′-CTCCTCCTTGTGGCTACCCT-3′, antisense: 5′-AGGCATGGGAACATTCCTGG-3′, IL-17 sense: 5′-AACCGATCCACCTCACCTTG-3′, antisense: 5′-TCTCTTGCTGGATGGGGACA-3′, β-actin sense: 5′-TGTTACAGGAAGTCCCTTGCCATC-3′, antisense: 5′-CTGTGTGGACTTGGGAGAGGAC-3′.

### 2.7. ELISA

HaCaT cells were stimulated with NiCl_2_ in 96-well dishes and culture supernatant was collected 24 and 48 h after the stimulation. The TNF-α and IL-1β production were measured using a human TNF-α ELISA (eBioscience, San Diego, CA, USA) and human IL-1β ELISA (Invitrogen, MA, USA) according to the manufacturers’ instructions.

### 2.8. Statistical Analysis

All experiments were repeated at least three times with similar results. Experimental values are given as means ± SD. The statistical difference was determined by a one-way ANOVA with LSD (least significant difference), Tukey’s HSD (honestly significant difference) test, and two independent samples: unpaired *t*-test or Pearson’s χ^2^-test. Statistical significance is presented in the following manner: * *p* < 0.05, ** *p* < 0.01, *** *p* < 0.001. The results of statistical analysis are shown in Supplementary Materials Tables S2 and S3.

## 3. Results

### 3.1. Profiles of Infiltrated Cells in Mucosa of OLP Lesions

**Figure 1:**
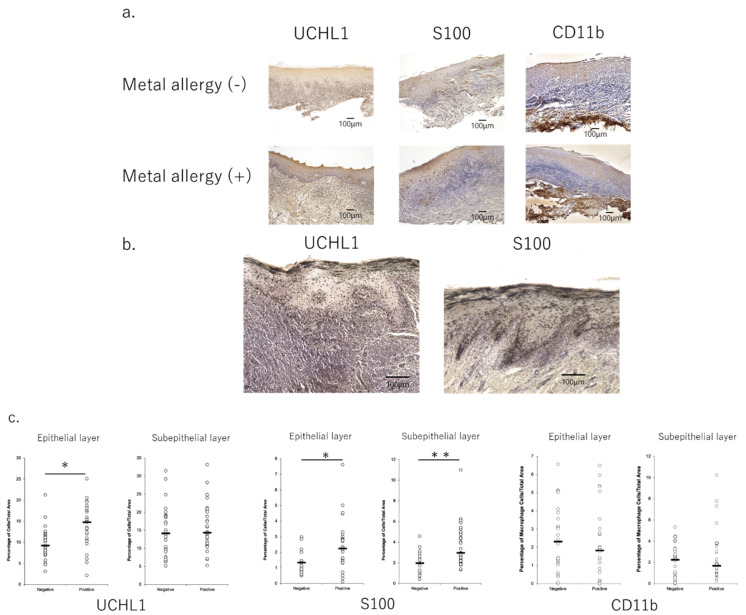
Profile of infiltrated cells in the mucosa of oral lichen planus lesions with immuno-histochemistry. (**a**) UCHL1 and S100 staining showed the difference between metal allergy-negative and -positive tissue. 100× magnification images. (**b**) UCHL1 and S100-positive cells in the epithelial tissues were more abundant in metal allergy-positive patients. Images with 200× magnification. (**c**) UCHL1 and S100-positive cells in the epithelial tissues were significantly more abundant in metal allergy-positive patients than in allergy-negative patients. Images were analyzed via Keyence Software. The tissue area was separated into epithelial and subepithelial areas with a border line. The percentage of cell number expressing each marker was calculated by dividing the total area of cells by the total area of the tissue, then converting to a percentage. Data are shown as mean ± SD. * *p* < 0.05, ** *p* < 0.01; unpaired *t*-test.

Supplementary Materials Table S1 shows the clinical information of the patients. There was no statistically significant difference in site of the lesion, types of prosthetic devices, and lesion-to-metal contact between positive and negative patients. Infiltration of immune cells, such as T cells, macrophages, and dendritic cells (DCs), into lesions was observed in all samples (Figure 1a). Moreover, the infiltration of DCs and T cells into epithelial tissue samples was significantly higher in patients who exhibited metal allergies than in those who did not (Figure 1b,c).

### 3.2. Pro-Inflammatory Cytokines Produced by Cultured HaCaT Cells Stimulated with NiCl_2_

**Figure 2:**
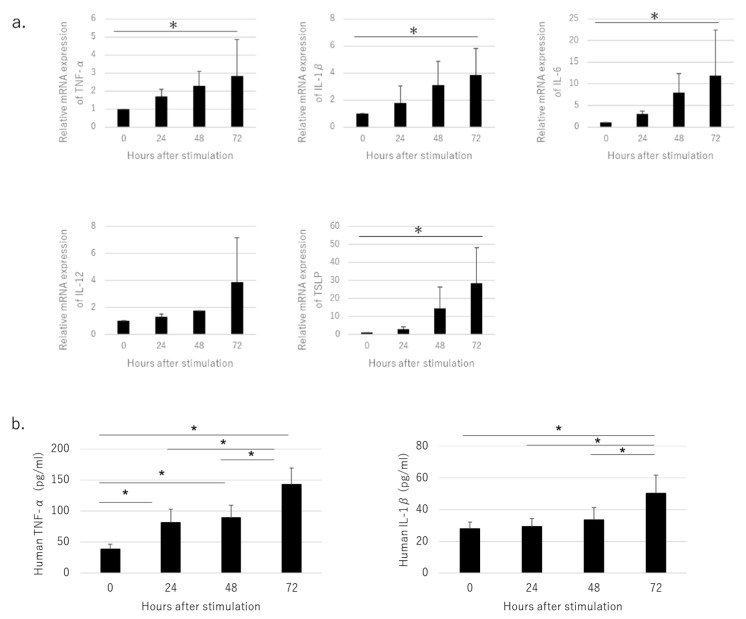
Pro-inflammatory markers produced by cultured HaCaT cells stimulated with NiCl_2_. (**a**) Expression of TNF-α, IL-1β, IL-6, and TSLP and IL-12 was increased in HaCaT stimulated with Ni, as measured by quantitative real-time PCR. (**b**) Production of TNF-α and IL-1β was increased in HaCaT cells stimulated with Ni. Left panel shows TNF-α production and right panel shows IL-1β production, as analyzed by ELISA. Data are shown as mean ± SD and are representative of at least three independent experiments. * *p* < 0.05; one-way ANOVA with LSD.

The expression of tumor necrosis factor (TNF)-α, interleukin (IL)-1β, IL-6, TSLP, and TSLPR mRNA was up-regulated 72 h after stimulation in a time-dependent manner (Figure 2a). Similarly, enzyme-linked immunoassay analysis of the culture supernatant of HaCaT stimulated with NiCl_2_ showed that the production of TNF-α and IL-1β both increased significantly after stimulation (Figure 2b).

### 3.3. Secretion of Pro-Inflammatory Cytokines in OLP Lesions

**Figure 3:**
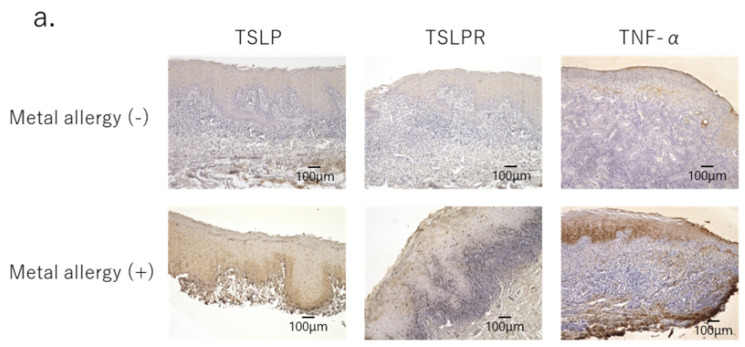

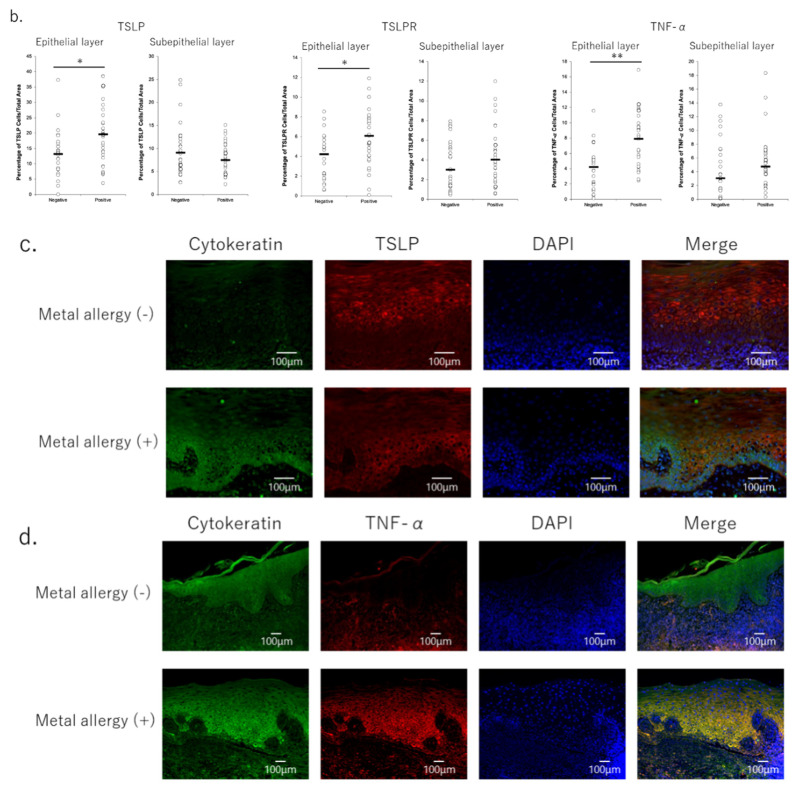
Pro-inflammatory cytokines secreted in oral lichen planus lesions. (**a**) TNF-α, TSLP, and TSLPR secretions were increased in the epithelial layer of oral lichen planus tissues in metal allergy-positive patients. Images with 100× magnification. (**b**) TSLP, TSLPR and TNF-α secretions were increased in the epithelial layer of oral lichen planus tissues in metal allergy-positive patients. * *p* < 0.05, ** *p* < 0.01; unpaired *t*-test. (**c**,**d**) TSLP and TNF-α secretions by keratinocytes were increased in the epithelial layer of oral lichen planus tissues in metal allergy-positive patients. (**c**) 200× magnification images, (**d**) 100× magnification images.

Although TSLP was produced in both allergy-negative and -positive patients, TSLP, TSLPR and TNF-α were all produced at significantly higher quantities in the mucosal epithelium samples of the former (Figure 3a,b). Immunofluorescence staining was used to identify the cells that produced these cytokines, revealing that TSLP was found to be produced by keratinocytes, especially in the basal layer of epithelium. Moreover, TNF-α expression was also widely observed in epidermal tissue samples (Figure 3d).

## 4. Discussion

OLP is a T-cell-mediated chronic inflammatory disease of unclear etiology that occurs in the oral mucosa. OLP commonly manifests in the buccal mucosa, tongue, and gingiva as white striations, erythema, or erosions. Microscopic examination of patients’ lesional tissue samples reveals a marked subepithelial band of lymphocytic infiltration, increased numbers of intraepithelial lymphocytes, and the degeneration of basal keratinocytes [8,9].

Although several factors have been suggested to be involved in the pathogenesis of OLP, the exact relationships among these factors remain to be elucidated. One leading hypothesis is that cellular immunity plays an important role in the pathogenesis of OLP. The degeneration of basal layer keratinocytes might plausibly be explained by a pathogenic process in which T cells accumulate and become activated in the basal keratinocytes of the epithelium, thereby damaging the keratinocytes. However, the antigen that attracts these T cells remains unclear. As both CD4^+^ T helper (Th) cells and CD8^+^ cytotoxic T cells are activated in OLP (B, C), there may not be one single antigen that triggers the disease. The majority of T cells adjacent to degenerated basal keratinocytes have been shown to be CD8^+^ T cells [8,9]. In addition, the number of Langerhans cells (LCs) showing enhanced expression of major histocompatibility complex class II was higher in OLP lesions. These activated LCs activate CD4^+^ T cells, which accumulate in the mucosal lamina propria and produce Th1-type cytokines such as IL-2 and interferon (IFN)-γ [8,9,10]. Metal allergy is an immune response that is initiated by the interaction of CD4^+^ cells with LCs and DCs. Although it is unclear whether OLP or metal allergy comes first, they may each mutually promote disease.

Wang et al. revealed that the balance between Th1 and Th2 cells plays an important role in the pathogenesis of OLP [10]. In this study, the expression of IFN-γ and IL-4 was found to be increased in the serum and lesional tissues of OLP patients compared with controls. Moreover, the IFN-γ/IL-4 ratio was significantly elevated in the peripheral blood samples of these patients, suggesting that Th1 cells may play a leading role in the Th1/Th2 immune balance postulated to have a role in the pathogenesis of OLP. Here, we found that metal allergy-positive patients with OLP produced more TNF-α in the epithelium than did negative patients, and that they exhibited a higher expression of TSLP in keratinocytes within the epithelial basal layer. TSLP is a cytokine that is produced by epithelial cells; it plays an important role in T cell differentiation by activating antigen-presenting cells and is also thought to be involved in the induction of Th2 cells by activating DCs and T cells. Its expression is regulated by many other cytokines, such as IL-1β and TNF-α [11,12]. Normal human bronchial epithelial cells cultured in the presence of IL-1β or TNF-α have been shown to exhibit increased mRNA expression of TSLP [11]. Here, the production of IL-1β and TNF-α increased over time when HaCaT was stimulated with Ni in vitro. This was followed by an increase in TSLP production, suggesting that these cytokines, which were produced by HaCaT itself, may induce the production of TSLP by HaCaT in an autocrine manner. The production of TSLP and TNFα by keratinocytes was also observed in the oral mucosal epithelium of patients with metal allergy-positive OLP; TSLP and TNFα production was significantly higher in these patients than in those who were negative for metal allergy.

In recent years, TSLP has been shown to be involved in the pathogenesis of allergic diseases such as asthma and atopic dermatitis, as well as inflammatory arthritis. Moreover, analysis conducted using genetically modified mice has revealed that TSLP is a master switch for the development of allergic diseases [13,14,15,16,17,18]. In particular, TSLP is known to be expressed in keratinocytes from patients with atopic dermatitis [13]. TSLP expression has also been revealed to be upregulated in the lesional epidermis of atopic dermatitis patients, compared to the levels found in the epidermis of healthy patients [13]. In mouse models, the presence of high levels of TSLP in the skin has been revealed to result in inflammatory disease, producing a predominance of Th2 cytokines. In addition, the expression of TSLP in the epidermis has been found to result in a spontaneous disease resembling atopic dermatitis. In this experimental model, elevated TSLP levels in the epidermis were shown to induce Th2 cytokine-associated inflammation. TSLP acts on CD11c^+^ DCs in the absence of IL-12, induces the OX40 ligand on DCs, and selectively induces naive T cells to differentiate into Th2 cells via OX40 on T cells [19]. Furthermore, TSLP is known to induce the differentiation of naive T cells into Th2 cells [19]. In addition, the expression of TSLP has been associated with the migration and activation of LCs in situ [13]. Based on these findings, TSLP produced by mucosal epithelial cells and skin keratinocytes that have been allergically injured by chemicals, microorganisms, or allergens could contribute to the induction of allergic inflammation by activating epidermal–dermal DCs [19]. Moreover, TSLP can directly promote Th2 cell differentiation in naive T cells via a combination of T-cell-receptor stimulation and TSLP treatment [20,21]. Interestingly, naive CD4^+^ T cells that have been treated with TSLP have been shown to exhibit the potential to induce IFN-γ, along with IL-4 and IL-2, within 48 h after treatment. This suggests that TSLP could also play a role in Th1-type inflammation [21].

Metal allergy is a Th1-type hypersensitivity reaction that involves CD11c^+^ DCs [22,23]. Thus, TSLP produced by keratinocytes may act on CD11c^+^ DCs to produce an allergic reaction due to metal stimulation. Ashrin et al. found that the expression of TSLPR on CD11c^+^ DCs was significantly increased in the ear skin of metal allergy mouse models, indicating that suppression of TSLP expression in the ear skin attenuated allergic inflammation [24]. Here, the numbers of DCs and T cells infiltrating the epithelium in OLP tissue were higher in metal allergy-positive patients than in -negative patients, and that the infiltrating DCs and T cells expressed TSLPR. In other words, TSLP-TSLPR signaling between keratinocytes and DCs, or between keratinocytes and T cells, was enhanced in the OLP lesions of metal-allergic patients.

Here, the stimulation of the mucosa with metal ions continued because mucosal tissue samples were collected before the patch test, prior to the removal of metals. Therefore, in the lesional mucosa of patients with OLP who were positive for metal allergy, the activation of TSLP-TSLPR signaling may have proceeded simultaneously due to both the pathogenesis of OLP and stimulation from oral metals.

The activation of keratinocytes and the increased expression of TSLPs stimulated by oral metals may increase the infiltration of T cells into the epithelium through interactions with TSLPR on T cells. This could explain why the number of T cells infiltrating the epithelium was found here to be higher in metal allergy-positive patients than in -negative patients. In addition, stimulation from metal residues within the oral cavity can infiltrate and activate LCs in the epithelium. Furthermore, TNF-α and IL-1β, which are produced by keratinocytes both in response to metal stimulation and during the pathogenesis of OLP, can also enhance the activation of LCs. Moreover, TNF-α also increases the expression of the vascular endothelial cell adhesion molecules E-selectin, ICAM-1, and VCAM-1, and it recruits lymphocytes to the mucosal lesion. TNF-α has been reported to be expressed in the epithelial basal layer, while TNF-R1 has been shown to be expressed in T cells that are infiltrating OLP areas [8,25,26,27,28]. Thus, the increased numbers of infiltrating DCs, LCs, and T cells in the OLP tissues of metal allergy-positive patients observed here may have occurred due to the pathology of metal allergy superimposing on that of OLP itself.

Considering the relationship between metal allergy and OLP, there are many similarities between the phenomena seen in OLP lesions and in metal allergic epithelium, suggesting that metal allergy may be an aggravating factor in the pathogenesis of OLP. In addition, the pathogenesis of OLP is multifactorial; thus, it is possible that metal allergy can increase the expression of TSLP in keratinocytes and that it can increase TNF-α levels in the epithelium. This would lead to the accumulation of T cells in the lesion site, fostering the formation of the disease. In addition, metals or metal-denatured proteins may be candidates as antigens for OLP.

Such a complex hypothesis for pathogenesis may be compared with the allergic march, in which patients with one allergic disease, such as atopic dermatitis, are affected by other allergic diseases, one after another. TSLPs have been suggested to be involved in the allergic march [15], but the precise nature of their involvement remains unclear.

A survey of metal allergies reported in 2015 revealed that in epidemiological studies, a high percentage of patients with metal allergies also had atopic dermatitis, allergic rhinitis, and food allergies [29]. Our finding that TSLP-TSLPR expression is significantly elevated in patients with OLP and metal allergies may be key to unravelling the connections between these conditions in future studies.

## 5. Conclusions

The infiltration of T cells into the epithelium was revealed here to be higher in the OLP lesions of metal allergy-positive patients than in those of metal allergy-negative patients. Moreover, TSLP-TSLPR signaling was higher in the epithelial tissue samples of the metal allergy-positive patients than in the metal allergy-negative patients. This signaling pathway between keratinocytes and T cells may be key to explaining the simultaneous development of multiple allergic conditions. Further studies on a larger scale are required to explore this proposed mechanism.

## Data Availability

All relevant data are included within the paper itself.

## Supplementary Materials

The following supporting information can be downloaded at: https://www.mdpi.com/article/10.3390/jcm11030519/s1, Figure S1: Image analysis with 100× magnification image; Table S1: Clinical information of patients. There were no statistically significant differences in clinical points between metal allergy-negative patients and positive patients; Table S2: Results of statistical analysis for Immunohistochemistry with SPSS. Table S3: Results of statistical analysis for clinical information of patients (Pearson’s χ^2^-test).
