# Supplementary material for: Metal Allergy Mediates the Development of Oral Lichen Planus via TSLP-TSLPR Signaling
Source: J Clin Med. 2022 Jan 20;11(3):519. doi: 10.3390/jcm11030519 (PMC8836592; doi:10.3390/jcm11030519)
Supplement: Supplementary file 1 [file jcm-11-00519-s001.zip › jcm-1523200-supplementary.pdf]

Subepithelial lesion   Epithelial lesion

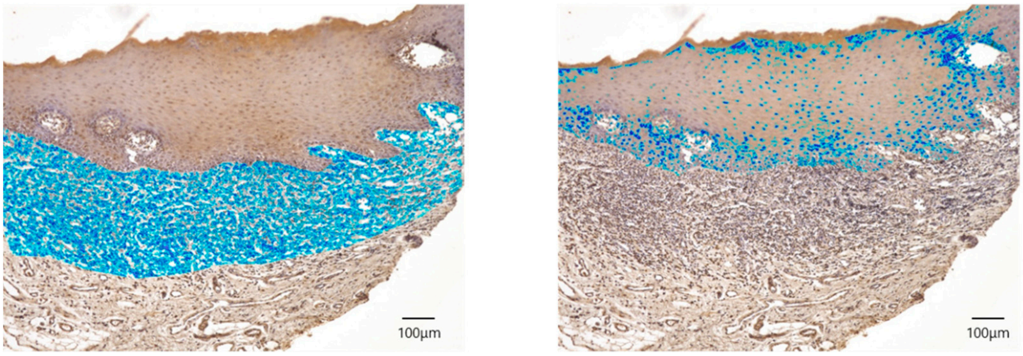

Figure S1. Image analysis with 100X magnification image. Immunohistochemistry images were taken by a Keyence All-in-One Fluorescence Microscope (BZ-X800, Osaka, Japan). Immunohistochemistry stained positive cells were detected as blue and the percentage of cell number was calculated by the total area of cell spreading divided total area of tissue by Keyence Software BZ-X800 Analyzer.

Table S1. Clinical information of patients. There were no statistically significant differences in clinical points between metal allergy-negative patients and positive patients.

Allergy	Negative	Positive
Sex	Male: 6, Female: 24	Male: 6, Female: 28
Average age	67.3	62.8
Lesion (number)	BM: 27, G: 13, T: 2	BM: 32, G: 8, T: 4
Prostesis (number)	Cr: 30, In: 13, AF: 6, PD: 4	Cr: 28, In: 7, AF: 1, PD: 4
Contact with metal prosthesis (cases)	23	29

Lesion: p = 0.337138464, Prosthesis: p = 0.282566, Contact: p = 0.336446589 (Pearson's χ^2-test). Lesion; BM: buccal mucosa, G: gum, T: tongue. Prosthesis; Cr: metal crown, In: metal inlay, AF: amalgam filling, PD: removable partial denture.

Table S2. Results of statistical analysis for Immunohistochemistry with SPSS.

UCHL1

Epithelial

Group Statistics

Groups		N	Mean	Std. Deviation	Std. Error Mean
UCHL1	Negative Samples	25	9.7516	3.85688	0.77138
	Positive Samples	27	14.1030	5.28896	1.01786

Independent Samples Test

		Levene's Test for Equality of Variances		t-test for Equality of Means					95% Confidence Interval of the Difference	
		F	Sig.	t	df	Sig. (2-tailed)	Mean Difference	Std. Error Difference	Lower	Upper
UCHL1	Equal variances assumed	2.909	0.094	-3.367	50	0.001	-4.35139	1.29253	-6.94752	-1.75526
	Equal variances not assumed			-3.407	47.476	0.001	-4.35139	1.27713	-6.91997	-1.78282

Subepithelial

Group Statistics

Groups		N	Mean	Std. Deviation	Std. Error Mean
UCHL1	Negative Samples	30	14.6385	6.73438	1.22952
	Positive Samples	31	15.5946	6.44927	1.15832

Independent Samples Test

		Levene's Test for Equality of Variances		t-test for Equality of Means					95% Confidence Interval of the Difference	
		F	Sig.	t	df	Sig. (2-tailed)	Mean Difference	Std. Error Difference	Lower	Upper
UCHL1	Equal variances assumed	0.124	0.726	-0.566	59	0.573	-0.95617	1.68800	-4.33384	2.42150
	Equal variances not assumed			-0.566	58.656	0.574	-0.95617	1.68921	-4.33669	2.42435

S100

Epithelial

Group Statistics

Groups		N	Mean	Std. Deviation	Std. Error Mean
S100	Negative Samples	15	1.4197	0.77126	0.19914
	Positive Samples	30	2.3925	1.54100	0.28135

Independent Samples Test

		Levene's Test for Equality of Variances		t-test for Equality of Means					95% Confidence Interval of the Difference	
		F	Sig.	t	df	Sig. (2-tailed)	Mean Difference	Std. Error Difference	Lower	Upper
S100	Equal variances assumed	3.041	0.088	-2.296	43	0.027	-0.97276	0.42370	-1.82723	-0.11829
	Equal variances not assumed			-2.822	42.987	0.007	-0.97276	0.34469	-1.66790	-0.27762

Subepithelial

Group Statistics

Groups		N	Mean	Std. Deviation	Std. Error Mean
S100	Negative Samples	17	2.0306	1.16723	0.28309
	Positive Samples	31	3.7049	1.99691	0.35866

Independent Samples Test

		Levene's Test for Equality of Variances		t-test for Equality of Means					95% Confidence Interval of the Difference	
		F	Sig.	t	df	Sig. (2-tailed)	Mean Difference	Std. Error Difference	Lower	Upper
S100	Equal variances assumed	3.074	0.086	-3.164	46	0.003	-1.67425	0.52918	-2.73944	-0.60906
	Equal variances not assumed			-3.664	45.738	0.001	-1.67425	0.45692	-2.59412	-0.75438

CD11b

Epithelial

Group Statistics

Groups		N	Mean	Std. Deviation	Std. Error Mean
CD11b	Negative Samples	26	2.4332	1.78015	0.34912
	Positive Samples	28	2.0854	2.00219	0.37838

Independent Samples Test

		Levene's Test for Equality of Variances		t-test for Equality of Means					95% Confidence Interval of the Difference	
		F	Sig.	t	df	Sig. (2-tailed)	Mean Difference	Std. Error Difference	Lower	Upper
CD11b	Equal variances assumed	0.132	0.718	0.673	52	0.504	0.34785	0.51711	-0.68981	1.38551
	Equal variances not assumed			0.676	51.909	0.502	0.34785	0.51483	-0.68528	1.38098

Subepithelial

Group Statistics

Groups		N	Mean	Std. Deviation	Std. Error Mean
CD11b	Negative Samples	30	1.9913	1.45560	0.26576
	Positive Samples	31	2.4376	2.35631	0.42321

Independent Samples Test

		Levene's Test for Equality of Variances		t-test for Equality of Means					95% Confidence Interval of the Difference	
		F	Sig.	t	df	Sig. (2-tailed)	Mean Difference	Std. Error Difference	Lower	Upper
CD11b	Equal variances assumed	1.836	0.181	-0.886	59	0.379	-0.44631	0.50347	-1.45375	0.56114
	Equal variances not assumed			-0.893	50.243	0.376	-0.44631	0.49973	-1.44992	0.55731

TSLP

Epithelial

Group Statistics

Groups		N	Mean	Std. Deviation	Std. Error Mean
TSLP	Negative Samples	24	13.3356	7.92342	1.61736
	Positive Samples	30	19.7892	9.70380	1.77166

Independent Samples Test

		Levene's Test for Equality of Variances		t-test for Equality of Means					95% Confidence Interval of the Difference	
		F	Sig.	t	df	Sig. (2-tailed)	Mean Difference	Std. Error Difference	Lower	Upper
TSLP	Equal variances assumed	1.612	0.210	-2.630	52	0.011	-6.45363	2.45381	-11.37757	-1.52969
	Equal variances not assumed			-2.690	51.968	0.010	-6.45363	2.39888	-11.26741	-1.63984

Subepithelial

Group Statistics

Groups		N	Mean	Std. Deviation	Std. Error Mean
TSLP	Negative Samples	30	9.6323	5.54505	1.01238
	Positive Samples	33	7.6066	3.38999	0.59012

Independent Samples Test

		Levene's Test for Equality of Variances		t-test for Equality of Means					95% Confidence Interval of the Difference	
		F	Sig.	t	df	Sig. (2-tailed)	Mean Difference	Std. Error Difference	Lower	Upper
TSLP	Equal variances assumed	2.748	0.103	1.767	61	0.082	2.02573	1.14624	-0.26631	4.31777
	Equal variances not assumed			1.729	47.125	0.090	2.02573	1.17182	-0.33150	4.38296

TSLPR

Epithelial

Group Statistics

Groups		N	Mean	Std. Deviation	Std. Error Mean
TSLPR	Negative Samples	24	3.9065	2.32540	0.47467
	Positive Samples	29	5.8614	2.85658	0.53045

Independent Samples Test

		Levene's Test for Equality of Variances		t-test for Equality of Means					95% Confidence Interval of the Difference	
		F	Sig.	t	df	Sig. (2-tailed)	Mean Difference	Std. Error Difference	Lower	Upper
TSLPR	Equal variances assumed	0.505	0.481	-2.693	51	0.010	-1.95487	0.72585	-3.41207	-0.49767
	Equal variances not assumed			-2.746	50.992	0.008	-1.95487	0.71182	-3.38392	-0.52582

Subepithelial

Group Statistics

Groups		N	Mean	Std. Deviation	Std. Error Mean
TSLPR	Negative Samples	28	3.5709	2.50705	0.47379
	Positive Samples	32	4.5999	2.97154	0.52530

Independent Samples Test

		Levene's Test for Equality of Variances		t-test for Equality of Means					95% Confidence Interval of the Difference	
		F	Sig.	t	df	Sig. (2-tailed)	Mean Difference	Std. Error Difference	Lower	Upper
TSLPR	Equal variances assumed	0.084	0.773	-1.438	58	0.156	-1.02901	0.71552	-2.46128	0.40326
	Equal variances not assumed			-1.455	57.933	0.151	-1.02901	0.70740	-2.44506	0.38704

TNF-α

Epithelial

Group Statistics

Groups		N	Mean	Std. Deviation	Std. Error Mean
TNFa	Negative Samples	24	4.1657	2.91191	0.59439
	Positive Samples	30	7.6760	3.41156	0.62286

Independent Samples Test

		Levene's Test for Equality of Variances		t-test for Equality of Means					95% Confidence Interval of the Difference	
		F	Sig.	t	df	Sig. (2-tailed)	Mean Difference	Std. Error Difference	Lower	Upper
TNFa	Equal variances assumed	0.668	0.417	-4.005	52	0.000	-3.51023	0.87641	-5.26887	-1.75158
	Equal variances not assumed			-4.077	51.753	0.000	-3.51023	0.86096	-5.23807	-1.78238

Subepithelial

Group Statistics

Groups		N	Mean	Std. Deviation	Std. Error Mean
TNFa	Negative Samples	28	4.2809	4.00448	0.75678
	Positive Samples	33	5.5107	3.79194	0.66009

Independent Samples Test

		Levene's Test for Equality of Variances		t-test for Equality of Means					95% Confidence Interval of the Difference	
		F	Sig.	t	df	Sig. (2-tailed)	Mean Difference	Std. Error Difference	Lower	Upper
TNFa	Equal variances assumed	1.055	0.309	-1.230	59	0.223	-1.22981	0.99966	-3.23012	0.77050
	Equal variances not assumed			-1.225	56.243	0.226	-1.22981	1.00421	-3.24129	0.78167

Table S3. Results of statistical analysis for clinical information of patients (Pearson's χ^2- test).

Lesion

	buccal mucosa	gum	tongue	
negative	27	13	2	42
positive	32	8	4	44
	59	21	6	86

	buccal mucosa	gum	tongue	
negative	28.9	10.3	2.9	42
positive	30.1	10.7	3.1	44
	59	21	6	86
				0.337138464

Prosthesis

	Cr	In	AF	PD	
negative	30	13	6	4	53
positive	28	7	1	4	40
	58	20	7	8	93

	Cr	In	AF	PD	
negative	33.6	11.4	4	4.6	53
positive	24.9	8.6	3	3.4	40
	58	20	7	8	93
					0.282566

Contact with metal prosthesis

	non-contact	contact	
negative	7	23	30
positive	5	29	34
	12	52	64

	non-contact	contact	
negative	5.6	24.3	30
positive	6.4	26.7	34
	12	52	64
			0.336446589
